# Dermoide du limbe au cours du syndrome de Goldenhar

**DOI:** 10.11604/pamj.2013.15.69.2844

**Published:** 2013-06-24

**Authors:** Zouheir Hafidi, Rajae Daoudi

**Affiliations:** 1 Université Mohammed V Souissi, service d'ophtalmologie A de l'hôpital des spécialités, Centre hospitalier universitaire, Rabat, Maroc

**Keywords:** Syndrome de Goldenhar, syndrome polymalformatif, malformation, Goldenhar Syndrome, polymalformative syndrome, malformation

## Image en médecine

Le syndrome de Goldenhar ou dysplasie oculo-auriculo-vertébrale est un syndrome polymalformatif rare, en rapport avec une anomalie de développement des premiers arcs branchiaux; il est le plus souvent unilatéral affectant les tissus mous et dans une moindre proportion le tissu osseux, associant de façon variable des malformations squelettiques, auditives et oculaires avec un retard mental. Son diagnostic est essentiellement clinique et relativement simple; l'atteinte ophtalmologique est assez caractéristique et représentée par le dermoide du limbe, à cette anomalie peut s'ajouter un colobome de la paupière, un syndrome de Duane et très rarement une microphtalmie ou une anophtalmie. Nous rapportons l'observation d'un nourrisson âgé de 2 années de sexe masculin, adressé dans notre formation dans le cadre du bilan général d'un syndrome poly malformatif associant un retard psychomoteur, hypoplasie du pavillon de l'oreille gauche associé à des appendices près auriculaires avec rétrognathisme. L'examen ophtalmologique note une formation tumorale blanchâtre de l’œil gauche, de localisation limbique temporale inférieure avec un fin duvet à sa surface évoquant un dermoide du limbe, la réfraction automatique n'a pas montré de vice réfractif, le reste de l'examen ophtalmologique est sans aucune anomalie, notamment il n'y a pas de déviation oculaire à l'examen oculomoteur. Devant ce tableau clinique le diagnostic de syndrome de Goldenhar a été retenu et l'abstention thérapeutique sur le plan ophtalmologique a été adoptée vu la bénignité de la lésion, et l'absence de vice réfractif qui pourrait être à l'origine d'une amblyopie anisométropique, auquel cas l'excision chirurgicale devient impérative.

**Figure 1:**
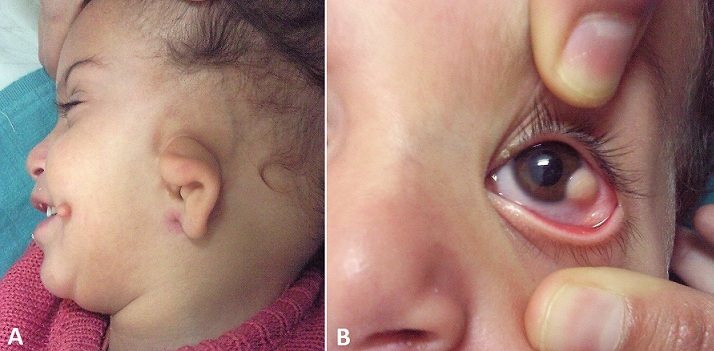
(A) appendice de la joue gauche avec un micro rétrognathisme et déformation du pavillon de l'oreille. (B) dermoide du limbe temporal inférieur de l’œil gauche

